# 

**Published:** 1908-03

**Authors:** 


Sixty-third Year.

Vol. LXIII. MARCH, 1908. No. 8

### BUFFALO MEDICAL JOURNAL

Established 1845 by AUSTIN FLINT M. D.

LIBRARY
SURGEON GENERAL'S OFFICE
MAR. 2- 1908

EDITOR:

WILLIAM WARREN POTTER, M. D.

ASSISTANT EDITORS:
~~WILLIAM~~ C. KRAUSS, M. D. NELSON W. WILSON, M. D.

ASSOCIATE EDITORS:
JAMES WRIGHT PUTNAM, M. D. ERNEST WENDE, M. D.
JOHN PARMENTER, M. D. JOHN A. MILLER, Ph. D.
MAUD J. FRYE, M. D.

